# Numerical Capacities as Domain-Specific Predictors beyond Early Mathematics Learning: A Longitudinal Study

**DOI:** 10.1371/journal.pone.0079711

**Published:** 2013-11-08

**Authors:** Vivian Reigosa-Crespo, Eduardo González-Alemañy, Teresa León, Rosario Torres, Raysil Mosquera, Mitchell Valdés-Sosa

**Affiliations:** 1 Department of Developmental Cognitive Neuroscience, Cuban Center for Neuroscience, Havana, Cuba; 2 Department of Education and Quality, Central Institute of Pedagogical Sciences, Havana, Cuba; University of Akron, United States of America

## Abstract

The first aim of the present study was to investigate whether numerical effects (Numerical Distance Effect, Counting Effect and Subitizing Effect) are domain-specific predictors of mathematics development at the end of elementary school by exploring whether they explain additional variance of later mathematics fluency after controlling for the effects of general cognitive skills, focused on nonnumerical aspects. The second aim was to address the same issues but applied to achievement in mathematics curriculum that requires solutions to fluency in calculation. These analyses assess whether the relationship found for fluency are generalized to mathematics content beyond fluency in calculation. As a third aim, the domain specificity of the numerical effects was examined by analyzing whether they contribute to the development of reading skills, such as decoding fluency and reading comprehension, after controlling for general cognitive skills and phonological processing. Basic numerical capacities were evaluated in children of 3^rd^ and 4^th^ grades (n=49). Mathematics and reading achievements were assessed in these children one year later. Results showed that the size of the Subitizing Effect was a significant domain-specific predictor of fluency in calculation and also in curricular mathematics achievement, but not in reading skills, assessed at the end of elementary school. Furthermore, the size of the Counting Effect also predicted fluency in calculation, although this association only approached significance. These findings contrast with proposals that the core numerical competencies measured by enumeration will bear little relationship to mathematics achievement. We conclude that basic numerical capacities constitute domain-specific predictors and that they are not exclusively “start-up” tools for the acquisition of Mathematics; but they continue modulating this learning at the end of elementary school.

## Introduction

The Number System [1,2] is thought to be responsible for the basic processing that engages mental representations of numerical quantities. According to current theories, the Number System constitutes a domain-specific cognitive scaffolding on which high-level mathematical competence is assembled [3,4]. Therefore, the relationship between basic numerical capacities (BNC) and subsequently developed mathematics skills has been of increasing interest. Up to now, most studies have applied cross-sectional designs that are useful in evaluating the associations between variables but not in drawing conclusions on the causality of the associations [5,6]. This limitation can be overcome by a longitudinal approach, which may help to clarify the direction of causal relationships for typical development, and may also highlight the role of early impairment in later development. Most longitudinal studies have, however, focused on the relationship between arithmetic and cognitive abilities (e.g., [7]), or have focused on a narrow age range, specifically early grades which is the crucial period when children acquire the first symbolic numbers and learn counting principles [4,8–12]. Consequently, there is insufficient evidence on the specific and unique role of BNC in middle and later stages of mathematics learning in which more sophisticated knowledge is acquired. 

As far as we know, two studies have focused on core indices of BNC and later mathematics skills with a longitudinal perspective. In the first one, the authors found that the numerical approximation ability of 14-year-old children correlated with the children’s past scores in standardized mathematics achievement tests, extending all the way back to kindergarten [13]. Moreover, this correlation remained significant when controlling for individual differences in a wide range of cognitive and performance factors. This finding strongly supports the domain-specific hypothesis on the role of BNC in mathematics attainment. However, owing to the retrospective design of this study, the direction of the causality is hard to define. Acuity in the mental representation of numerical magnitudes at age 14 might be the cause, but it can also be the consequence of mathematics proficiency (see [14] for a comprehensive analysis). In the second study, the authors reported the first longitudinal research that prospectively tracks children’s core numerical competencies and arithmetic development throughout all elementary school years [15]. The participants were grouped according to individual reaction time (RT) for numerical comparison and subitizing, as slow, medium and fast performers. The authors found that subgroup classification at 6 years of age predicted computation ability at 6 years, 9.5 years, and 10 years. In a separate analysis, they also found that the subgroups did not differ in processing speed or nonverbal reasoning and concluded that subitizing and number comparison do not tap general cognitive abilities but reflect individual differences that are specific to the domain of numbers. 

Despite the relevance of these findings, this study failed to show evidence supporting the domain-specific hypothesis because the effects of the core BNC in the presence of the domain-general abilities were not extensively analyzed. However, this kind of stringent analysis is essential in testing whether the BNC measures retain their status as unique predictors. This point is critical because several studies have revealed the predictive power of the domain-general mechanisms (e.g., speed of information processing, working memory and logical reasoning) in solving arithmetic problems (e.g., [16–19]). Nevertheless, except for one [19] none of them have assessed these potential mechanisms simultaneously with one another and with domain-specific measures of BNC. 

Given the limited evidence available and the need for further testing of the domain-specific hypothesis within a longitudinal approach, here we designed a one year follow-up study to evaluate whether measures of BNC are related to individual differences in more sophisticated mathematics skills at the end of elementary school after controlling for domain-general cognitive abilities. This design allowed us to evaluate the domain-specific role of BNC on mathematical learning above and beyond early stages of learning.

 As a further step toward this goal, we have tried to address some methodological issues presented in previous developmental studies. Firstly, the multi-componential nature of mathematics was taken into account to design outcome measures. As a consequence, we can explore the relative contributions of BNC and domain-general mechanisms in relation to the nature of mathematics performance (e.g., [19]). Secondly, we considered the assessment of mathematics achievement with a time limit for performance. Under this condition, we can differentiate children who efficiently process numerical information from those who do not [20]. Moreover, previous reports revealed that BNC accounted for individual differences in performance with timed mathematics tests better than with untimed mathematics tests [8,21]. Thirdly, we focused on RT and accuracy trade-off analysis (efficiency) instead of response accuracy in BNC tasks because several developmental studies often needed to deal with ceiling effects from a certain age onward (e.g., [22]). Efficiency-based measures also allow us to inspect developments in the processing of numerosities and magnitudes and enable us to observe developments of standard effects of number processing (e.g., numerical distance effect) that are consistently reported in adults [21,23]. These effects could be more informative on how numbers and magnitudes are represented and processed in the cognitive system than the accuracy measures. 

Considering these methodological issues, in the present research the measurement of BNC was focused on the size of well-known effects of basic numerical processing based on a RT-accuracy trade-off analysis and the measurement of mathematics outcomes relied on multi-component and speeded tests. This design could reveal a more fine-grained picture of the relationship between the distinctness of quantity representations and individual differences in performance of diverse components of mathematics knowledge in later elementary education.

At the beginning of the study we assessed BNC in 3^rd^ and 4^th^ graders using two RT-based tasks that required the understanding of numerosity and the ability to recognize and judge small numerosities (up to 9), which required minimal cognitive resources and low levels of formal mathematics achievement. We therefore used a simple number comparison, which would test whether children understood number magnitudes, and a simple enumeration task. Similar tasks had been extensively reported in previous developmental studies (see [24] for a review). As pointed out, we focused on the size of numerical effects elicited by these tasks because they provide indicators for the preciseness of representations of numerical magnitudes. Such is the case of the numerical distance effect: when adults and school-aged children compare numerical stimuli for their relative magnitude, they are faster and more accurate at making responses when the numerical distance separating two numbers is relatively large than when it is small [21,25]; and the subitizing and counting effects: when sets are enumerated and participants are faster and more accurate on set sizes of 1-3 or 4 than on set sizes of 5 or larger [8,15,25,26]. 

As stated above, the potential relationships between the numerical effects and mathematics achievement could be related to more general cognitive differences between individuals. Such general cognitive differences would presumably be related to both mathematical skills and other crucial cognitive abilities such as reading. We therefore also collected measures of the children’s nonverbal reasoning, processing speed and reading decoding to help assess the specificity of the relationships between BNC and mathematics achievement and to rule out that the possible relationships may be due to individual differences in more general cognitive processes, rather than to individual differences in the processing and representation of numerosities.

One year later, several components of mathematics achievement were evaluated using two timed tests. One test was based on the mathematics curriculum for the grade and the other on fluency in solving simple calculations. We focused on fluency because it has been identified as a core arithmetic skill [27]. Moreover, attaining calculation fluency is difficult for many children (e.g., [28]). In addition, reading fluency and reading comprehension were assessed as outcomes of reading achievement.

In summary, we had three aims. The first was to investigate whether the numerical effects are domain-specific predictors of mathematics development at the end of elementary school by exploring whether they explain additional variance of later mathematics fluency after controlling for the effects of other cognitive skills, focused on nonnumerical aspects. Our second aim was to address the same issues but applied to achievement in the mathematics curriculum that requires solutions to fluency in calculation. These analyses assess whether the relationship found for fluency generalize to mathematics content beyond fluency in calculation. As a third aim, the domain specificity of the numerical effects was examined by analyzing whether they contribute to the development of reading skills, such as decoding fluency and reading comprehension, after controlling for general cognitive skills and phonological processing. 

## Methods

### Ethics statement

The study was approved by the Institutional Review Board of the Cuban Center for Neuroscience. Written consent was obtained from all parents, and all participants provided verbal assent for all assessments.

### Participants

A total of 49 children of grades 3^rd^ (n= 16, 7 boys and 9 girls, mean age= 9.3, SD= 0.43; mean of raw scores of Ravens Colored Progressive Matrices (CPM) = 22.5, SD =5.15), and 4^th^ (n= 33, 13 boys and 20 girls, mean age= 10.05; SD= 0.48; mean of Raven CPM (raw score) = 23, SD= 5.3) participated in the study. Children were recruited from an elementary school in Marianao, an urban municipality of Havana City, and were followed-up for one year, until reaching 4^th^ and 5^th^ grades, respectively. 

### Tests of the first point of the study (T_1_)

#### Ravens CPM test [29]

This test was administered as a measure of nonverbal reasoning ability. In this test, a colored pattern is shown with a missing piece. Below the pattern, six pieces, all fitting in the blank but with different patterns, are shown. The child has to select the piece that fits in the pattern above. The total number of correct selections was recorded. Each child completed the entire test, consisting of 36 items. 

#### Basic numerical battery (BNB) [23]

This battery comprises item-timed computerized tests, with a structure similar to that of the *Dyscalculia Screener* [30]. BNB includes a simple reaction task and two numerical capacity tests: Dot Enumeration and Symbolic Comparison. Each test includes practice trials to ensure that instructions were understood. The response is made by pressing the corresponding key in the numeric pad (right side of the keyboard). RT and errors are recorded. The critical point is that both, speed and accuracy will index capacity. RTs are removed in trials in which the children responded incorrectly.

1. Simple reaction time.

Children were asked to press the space bar as soon as they saw a square in the center of the display. The inter-stimulus presentation time was variable (500 -1,500 ms). This was considered a baseline measure for processing speed. Twenty trials were presented. Five practice trials were presented before starting the test.

2. Dot enumeration.

Groups of randomly arranged dots ranging from 1 to 9 were presented on the computer. Children were asked to enumerate the sets and to respond as quickly as they could without making any mistakes. Stimuli remained on the screen until the children responded. RT s and errors were recorded by pressing the key corresponding to the number of dots enumerated. There were altogether eighteen trials, with each number from 1 to 9 being represented twice in a pseudo-random order with the proviso that no item occurred twice in succession. Five practice trials were presented before starting the test. We assume that enumeration involved two strategies: subitizing for numerosities four or fewer, counting for four or more, and that there would be individual variations in the combination of strategies depending on both the individual’s numerical capacity, age, experience with counting, and so on.

3. Symbolic comparison.

Children were presented with two digits (1-9) on the computer, one to the left and one to the right of the screen and they were asked to compare the magnitude of the numbers from left to right (e.g., “5 is less than 7”, “7 is greater than 5”). The numerical distance between stimuli ranged from 1 to 3, with 12 comparison trials per distance. The trials were presented in a pseudo-random order. Children were asked to respond using a three-choice selection. Choices were presented in the lower part of the screen simultaneously with the stimuli and were expressed in the following manner:”press 1 for less than”, “press 2 for equal to” and “press 3 for greater than”. 

#### Word and pseudoword reading

This is a test included in the SAL battery [31]. The children were required to read 60 words balanced by frequency, number of letters and syllables and 30 pseudowords. Each stimulus was presented sequentially in white, Arial size 12 letter, centered on a black background on the computer. The trial ended after the child responds or after 5000 ms with no response. Ten practice trials were presented initially to ensure that the children understood the task. Responses were verbal and triggered a voice-activated key which measured reaction latencies from the onset of presentation. Errors were recorded by the experimenter. 

### Variables calculated for tests of T_1_

#### Numerical effect variables

These variables were calculated to quantify individual differences in the size of typical effects elicited when some kind of numerical information is processed. Such is the case of the numerical distance effect occurring when numerical magnitudes are compared and the subitizing and counting effects that are elicited by enumeration of sets. Typically, researchers compute the individual slopes based on RT to capture these effects. The decreases in slopes are generally interpreted as improvements in the efficiency of numerical processing [21,25,32]. For a more direct approach to this interpretation, we decided to evaluate individual differences in numerical effects using efficiency measures (EM) instead of RTs.

To calculate the numerical distance effect index (NDE), individual values of EM were obtained primarily for numerical distances 1 and 3. Each EM was computed using the median of the correct RT divided by the proportion of hits. This is an inverse measure; lower numbers indicate better performance. EM could be considered as an adequate descriptor of the speed-accuracy trade-off [30,33]. To quantify individual differences in the size of the NDE, we used a similar formula to that proposed by Holloway and Ansari [21]. We subtracted the EM for distance 3 from the EM for distance 1. This value was then divided by the EM for distance 3 for each child, thereby yielding a measure of the increase in efficiency from small to large distances while accounting for the individual differences in efficiency; hence, the greater the increase in the value of the NDE index, the larger the size of this effect. Individuals with larger distance effects are thought to have less distinct representations of numerical magnitude [21]. The NDE values were distributed normally with a skew value of 1.65 (SE=.34, 95% CI= .53). 

To calculate the subitizing effect index (SE) and the counting effect index (CE), individual values of EMs were obtained primarily for numerosities 1 and 3, and 5 to 8. The formula proposed by Holloway and Ansari [21], was extrapolated to yield a measure that also quantifies the changes in efficiency from small to large sets in the enumeration task. Specifically, the SE was computed using the EMs for numerosities 1 and 3. To quantify individual differences in the size of the SE, we subtracted EM for numerosity 1 from EM for numerosity 3. This value was then divided by the EM for numerosity 1 for each child to yield a measure of the minimal and nonlinear variation in efficiency from 1 to 3 numerosities while accounting for the individual differences in efficiency. Consequently, the closer the value of the SE is to zero; the more efficient the mechanism of subitizing. The SE values were distributed normally with a skew value of .90 (SE=.34 95% CI= .23). 

The EMs for numerosities 5 to 8 were used to calculate the CE index. To quantify individual differences in the size of the CE, we subtracted EM for numerosity 5 from EM for numerosity 8. This value was then divided by the EM for numerosity 5 for each child to yield a measure of the decrease in efficiency from small to large numerosities while accounting for individual differences in efficiency. The formula expresses that the greater the increase in the value of the CE index, the larger the size of this effect. Notice that the expected linear increases in EM for numerosities 5 to 8 are related to values of CE close to 1. So, individuals with larger CE are less efficient in counting. The CE values were distributed normally with a skew value of 2.02 (SE=.34 95% CI= .82).

The total score (correct responses) was calculated for Raven CPM. Also, EMs of word and pseudoword reading were obtained using the median of RT divided by the proportion of hits in each case.

### Tests used in the second point of the study (T_2_)

#### Mathematics fluency

A sheet with 100 basic exercises of addition, subtraction and multiplication, including numbers from 1 to 9, were presented to the children in a combined form. Calculations requiring the use of the rules of 0 (e.g., 2 +0) were not included. The children were asked to make as many calculations as they could in 3 minutes following the numerical order of the columns on the sheet. Internal consistency reliability was .95

#### Mathematics curriculum-based measures (CBM)

Two tests based on mathematics curriculum were designed for 4^th^ and 5^th^ grades, respectively. Each test consisted of four sets of exercises with time constriction by set: Set I. Numeration: Six problems dealing with writing numerals and numbers, rounding and determining successor and predecessor. Set II. Measurement: Six problems for converting magnitudes (e.g., “A container with oranges weighed 3260g. This weight converted to kg is ___”). Set III. Arithmetic: Twelve computational problems (e.g., “$\frac{3}{5}+\frac{1}{3}$”; “13881:34”); and Set IV. Word Problems that include four exercises (e.g., “A truck is moving at a speed of 300 meters per minute. How many kilometers will it cover in one hour?”). Both tests were constructed by selecting problem types representing a proportional sampling of the mathematics skills within the national curriculum. Coefficient alpha was .91.

#### Reading fluency

This test is an adaptation of the Silent Contextual Reading Fluency test (TOSCRF) [34]. Children were presented with short passages formed by rows of contextually related words, ordered by reading difficulty; all words were printed in uppercase without any spaces or punctuation between the words (e.g., AYELLOWBIRDWITHBLUEWINGS). Children were asked to draw a line between the boundaries of as many recognizable words as possible within 3 minutes (e.g., A/YELLOW/BIRD/WITH/BLUE/WINGS). The passages became gradually more complex in content, vocabulary, and grammar (embedded phrases, sequenced adjectives, affixes, etc.).

#### Reading comprehension

This is a test included in the SAL battery [31] was used to measure reading achievement. The children were asked to read a grade adjusted text. The text was presented in white, Arial size 12 letter, centered on a black background on the computer screen. After reading, the children were asked to respond true or false to ten propositions referring to the text by pressing the arrow keys (left for True and right for False). The children could not refer to the text for the entire duration of the comprehension task. The questions assessed both literal (e.g., fact-finding, ordering information) and inferential (e.g., deriving word meaning and making inferences beyond sentence level) text comprehension skills. A score was given for each correct answer. The overall reading time and total comprehension scores were calculated. 

### Variables calculated for tests of T_2_

Total scores were calculated for the Mathematics CBM, the Mathematics Fluency and the Reading Fluency tests. A measure was obtained from the Reading Comprehension test dividing the average reading time per decoded word by the comprehension score. 

### Procedure

Tests corresponding to T_1_ were administered in April to the children enrolled in 3rd and 4th grades. The assessment was conducted in a quiet and illuminated room within the school. Each child was evaluated individually in a single session that lasted from 20 to 30 min. When evaluating BNC, the child sat next to the experimenter in front of a computer. A Toshiba Satellite laptop P4 - M 1.9 GHz - 15" TFT connected to a conventional alphanumeric keyboard was used. 

Tests corresponding to T_2_ were administered one year later, between April and May, while the children were in 4^th^ and 5^th^ grades. The Mathematics CBM, the Mathematics Fluency and the Reading Fluency tests were group-administered. The Reading Comprehension test was individually administered. All tests were administered individually to children who had moved to other schools.

## Results

The means and standard deviations of all measures are shown in Table 1. Results of the tasks in T_1_ and T_2_ show considerable individual differences in the children’s predictors and outcomes.

**Table 1:** Means and standard deviations for all measures.

	T_1_		T_2_	
	3rd grade	4th grade	4th grade	5th grade
*General Predictors*				
Age	9.3 (.42)	10.5 (.48)	__	__
Nonverbal reasoning	22.5 (5.1)	22.9 (5.2)	__	__
Processing Speed	480.8 (120.7)	444.4 (115.6)	__	__
*Reading Predictors*				
Lexical processing	1844.1 (344.6)	1488.3 (403.1)	__	__
Phonological processing	2275.3 (414.4)	1950.7 (572.4)	__	__
*Numerical Predictors*				
Size of NDE	.489 (.56)	.379 (.27)	__	__
Size of Subitizing Effect	.324 (.2)	.262 (.16)	__	__
Size of Counting Effect	.767 (.55)	.682 (.28)	__	__
*Outcomes*				
Mathematics Fluency	__	__	35.06 (14.4)	50.6 (21.6)
Mathematics CBM	__	__	11.18 (5.06)	10.27 (5.8)
Reading Fluency	__	__	47.6 (19.5)	59.5 (33.9)
Reading Comprehension	__	__	9.87 (4)	12.75 (5.1)

Note. Standard deviations are in parentheses. NDE: Numerical Distance Effect. Mathematics CBM: Mathematics Curriculum-Based Measures.

To provide more detailed information on the children’s task performance for critical numerical predictors, a series of mixed-design analyses of variance (ANOVA) were performed for Dot Enumeration and Symbolic Comparison tasks. 

The expectation that children would subitize quantities up to 3, and count for 4 to 8 was supported by EM data. EMs were analyzed in a 2 (grade: 3^rd^, 4^th^) x 8 (numerosities: 1 to 8) ANOVA. Because the assumption of sphericity was violated, the within-participants effect and interactions are reported using the Greenhouse–Geisser adjustment. Means and standard errors of the EMs by numerosity and by grade can be found in Table 2.

**Table 2:** Means and standard errors of efficiency measures for each level of numerosity and distance.

Dot Enumeration Task			Symbolic Comparison Task		
Numerosity	3^rd^ grade	4^th^ grade	Distance	3^rd^ grade	4^th^ grade
1	1368 (59)	1341 (41)	1	3260 (245)	2276 (170)
2	1518 (60)	1380 (42)	2	3052 (240)	2136 (167)
3	1777 (72)	1665 (50)	3	2359 (160)	1647 (111)
4	2818 (161)	2449 (112)	__	__	__
5	3106 (151)	2974 (105)	__	__	__
6	3923 (173)	3725 (120)	__	__	__
7	4531 (194)	4389 (135)	__	__	__
8	5090 (224)	4645 (156)	__	__	__

Note. Standard errors are in parentheses.

We found that the main effect of grade was nonsignificant but the main effect of numerosity was highly significant (*F*(3.49, 164.16) = 395.02, p<.001, power= .99). In addition, we did not find a significant Numerosity x Grade interaction. According to post hoc Bonferroni-corrected *t* tests (α= .05/120 = .0041) the children’s EMs on numerosities of one, two and three did not differ at either grade level. Differences were, however, significant for numerosities of four to eight. In previous studies the subitizing range had been variably defined, sometimes ranging to three [8,26,33,35,36] and sometimes to four [14,37]. As the current study involved children who might have a comparably restricted subitizing range, we define the subitizing range as the numerosities one to three. Numerosity four could not be clearly ascribed to either the subitizing or the counting range and was thus excluded from the statistical analysis. Furthermore, as previous studies (e.g., [37]) have demonstrated, end effects for the enumeration of the largest numerosity of a set, the 9-dot stimuli were also excluded from the statistical analysis and the counting range was determined as numerosities five to eight. Based on these results, the size of subitizing and counting effects were calculated to quantify individual differences as was described previously in the Method section.

Next, an ANOVA was conducted using EM for each numerical distance (three levels: distances 1–3) as the within-participants variable, and grade (two levels: 3^rd^ - 4^th^) as the between-participants variable. In this case, the sphericity assumption was confirmed. Mean and standard error of the EM for each numerical distance by grade are shown in Table 2.

We found a significant main effect of distance, with the small distance having longer EM than the large distance ( F(2, 94) = 23.20, p < .001, power =.99 ). More specifically, Bonferroni corrected t tests (α= .05/3 = .16) demonstrated that the comparison with Distance 1 was more efficient than with distance 3; but EMs were nonsignificantly different for Distances 1 and 2. We also found a main effect of grade (F(1, 94) = 14.39, p < .001, power = .99), which reflects the finding that 4^th^ graders were significantly faster than 3^rd^ graders. Finally, we did not find a significant interaction between Grade and Distance. Based on these results, the size of NDE was calculated to quantify individual differences in symbolic comparisons as was described in the Method section.

**Figure 1:**
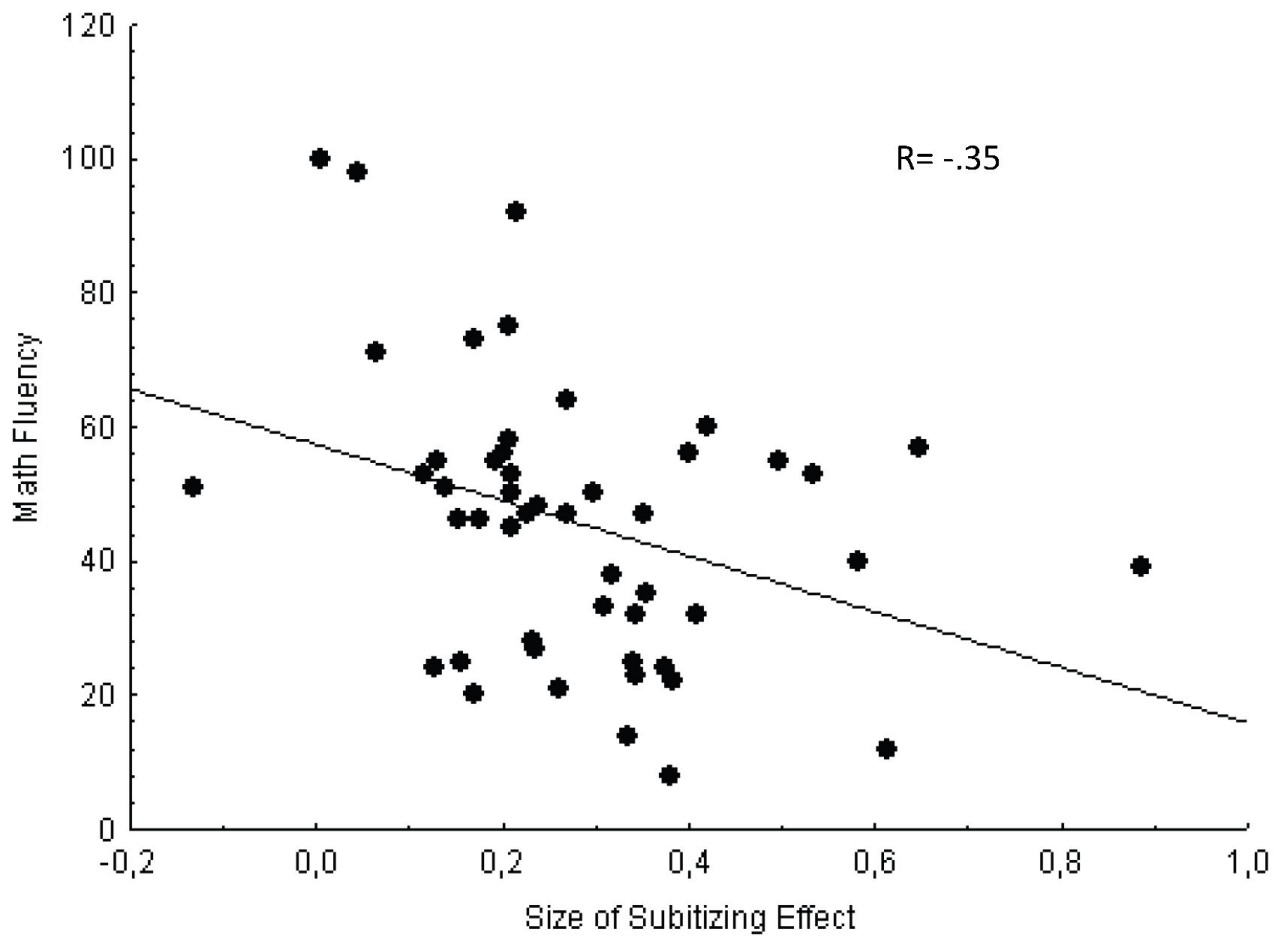
Scatterplot showing significant correlation between mathematics fluency and the size of the subitizing effect. The solid line represents the linear regression for this relationship.

Although we are testing directional hypotheses, significance values of all correlational analyses come from a two-tailed distribution. As shown in Table 3, a significant association occurred between all outcome measures (R: .38 to .69, p<.01, power: .78 to .99). Intellectual ability was significantly correlated with the outcome measures, indicating that children who were in general better in nonverbal reasoning also showed better performance in reading and mathematics (R: .28 to .65, p<.05, power: .50 to .99). However, the numerical effect indexes were not significantly correlated among themselves or with intellectual ability. Furthermore, the numerical effects were not related to any of the outcome measures, but the size of the subitizing effect was related to the mathematics fluency score (R= -.35, p<.05, Power= .71). Figure 1 shows this significant association. Note that the children who exhibited a larger subitizing effect (SE values close to zero) showed, one year later, higher scores in mathematics fluency. In contrast, efficiencies on lexical and phonological processing were significantly associated (R=.84, p<.001, Power= .99) and were also associated to reading outcome measures (R: -.37 to -.48, p<.01, power: .76 to .95).

**Table 3:** Correlations between predictors (T_1_) and Mathematics and Reading outcomes (*T*_*2*_).

	*Variables*	1	2	3	4	5	6	7	8	9	10	11
	**Outcomes**											
1	Mathematics Fluency	-	.466^**^	.49^***^	.48^***^	.371^**^	-.34^*^	-.28	-.22	-.23	-.35^*^	.25
2	Mathematics CBM		-	.69^***^	.38^**^	.65^**^	-.34^*^	-.22	-.16	-.18	-.17	.19
3	Reading Fluency			-	.67^***^	.52^***^	-.26	-.39^**^	-.25	-.11	-.15	-.06
4	Reading Comprehension				-	.28^*^	-.10	-.48^***^	-.37^**^	-.13	-.07	-.06
	**Predictors**											
5	Nonverbal reasoning					-	-.25	-.16	-.09	-.08	.12	.23
6	Processing speed						-	.16	.03	.27	.07	-.15
7	Lexical processing							-	.84^***^	.05	-.01	.11
8	Phonological processing								-	.01	-.09	.06
9	Size of NDE									-	.02	-.02
10	Size of Subitizing Effect										-	-.08
11	Size of Counting Effect											-

Note. NDE: Numerical Distance Effect. Mathematics CBM: Mathematics Curriculum-Based Measures.

* *p*<0.05 **p<0.01***p<0.001

Hierarchical regression analyses were conducted to investigate up to what extent the mechanisms involved in subitizing, counting and comparing numerical magnitudes explain unique variance in mathematics and reading achievement. Eight steps (see Table 4 and 5) were sequentially included in these analyses to determine whether the size of the numerical effects explained variance within two measures of mathematics outcome and two measures of reading outcome above and beyond general predictors: age (Step 1), nonverbal reasoning (Step 2), general processing speed (Step 3); and cognitive capacities that might also be reflected in other cognitive skills such as reading: lexical (Step 4) and phonological (step 5) processing. Predictor variables were entered in a fixed order to provide the most stringent test for the roles of variables predicting reading and mathematics skills after controlling for the effect of prior general cognitive skills. The constant order of entry was based on the outlined predictions and predictive correlations. 

**Table 4:** Hierarchical Regression Models predicting mathematics fluency and curriculum competence from numerical effect indexes.

		Mathematics Fluency			Mathematics CBM		
		β(standard)	ΔR^2^	F(change)	β(standard)	ΔR^2^	F(change)
	*General Predictors*						
1	Age	.36	.133	7.050^**^	-.03	.001	.066
2	Nonverbal reasoning	.32	.107	6.307^*^	.68	.459	38.333^***^
3	Processing speed	-.24	.055	3.429	-.17	.029	2.521
	Total ΔR^2^		.295			.489	
	*Reading Predictors*						
4	Lexical processing	-.13	.017	1.046	-.13	.017	1.458
5	Phonological processing	.32	.029	1.841	-.04	.000	.040
	Total ΔR^2^		.046			.017	
	*Numerical Predictors ^a^*						
	Size of NDE	-.09	.008	.507	-.12	.013	1.116
	Size of Counting Effect	.25	.055	3.707^†^	.01	.000	.022
	Size of Subitizing Effect	.29	.071	5.254^*^	-.33	.095	9.687^**^
	Total ΔR^2^		.134			.108	

Note. ^a^ Indicates the values of standardized beta coefficients, incremental R^2^ and F obtained for each numerical predictors when was added in step 8. NDE: Numerical Distance Effect. Mathematics CBM: Mathematics Curriculum-Based Measures.

* p<.05 **p<.01 ***p<.001 ^†^ p=.061

**Table 5:** Hierarchical Regression Models predicting reading achievement from numerical effect indexes.

		Reading Fluency			Reading Comprehension		
		β(standard)	ΔR^2^	F(change)	β(standard)	ΔR^2^	F(change)
	*General Predictors*						
1	Age	.12	.017	.774	.08	.007	.303
2	Nonverbal reasoning	.51	.263	16.407^***^	-.27	.075	3.688^†^
3	Processing speed	-.12	.015	.941	.02	.001	.035
	Total ΔR^2^		.295			.083	
	*Reading Predictors*						
4	Lexical processing	-.30	.082	5.661^*^	-.45	.189	11.136^**^
5	Phonological processing	.16	.008	.536	.08	.002	.118
	Total ΔR^2^		.090			.191	
	*Numerical Predictors ^a^*						
	Size of NDE	-.02	.001	.041	-.11	.012	.699
	Size of Counting Effect	-.02	.000	.032	-.06	.004	.207
	Size of Subitizing Effect	-.22	.046	3.146	-.14	.017	.950
	Total ΔR^2^		.047			.026	

Note. ^a^ Indicates the values of standardized beta coefficients, incremental R^2^ and F obtained for each numerical predictors when was added in step 8. NDE: Numerical Distance Effect.

* p<.05 ***p<.001 ^†^ p=.063

Mathematics Fluency and Mathematics CBM were defined as outcomes in separate analyses (power .82 to .84). In a model, the numerical predictors NDE, CE and SE were sequentially added in steps 6, 7 and 8 to determine whether individual differences in SE could explain variance over and above general predictors, NDE and CE. In a complementary set of analyses, steps 6, 7 and 8 were switched to determine the specific contribution of NDE and CE in explaining variance over and above general predictors and the other numerical predictors. The values of standardized beta coefficients, incremental R^2^ and F obtained for all variables included in the models are shown in Table 4. Note that the total amount of the variance explained by general, reading and numerical factors in predicting Mathematics Fluency and Mathematic CBM were 47.5% and 61.4%, respectively. The hierarchical regression analyses showed that the size of SE was a domain-specific predictor of both mathematics outcomes assessed one year later. In fact, after controlling for other variables, the size of SE significantly predicted 7.1% of the variance observed in mathematics fluency and 9.5% of individual variability in the achievement in mathematics curriculum. The size of CE explained a marginally significant amount of unique variance in fluency (5.5%) and the size of NDE did not significantly contribute to explaining individual variability in the measures of later mathematics achievement. Furthermore, the reading predictors did not explain additional variance of later mathematics skills after controlling for the effects of general cognitive skills.

Complementary analyses were run to test the domain specificity of the numerical predictor variables. In this case, hierarchical regressions were run following the same procedure described above. Here, Reading Fluency and Reading Comprehension were defined as outcomes in separate prediction models (statistical power .43 and .25, respectively). As shown in Table 5, the general cognitive, numerical and reading predictors explained 43.2 % of the variance in Reading Fluency and 30% of the variance in Reading Comprehension. Note that the numerical predictors did not significantly contribute to explaining individual variability in reading skills. However, the efficiency in lexical decoding explained a significant amount of unique variance found for Reading Fluency and Reading Comprehension (8.2% and 19.8%, respectively). Interestingly, the efficiency in phonological processing did not significantly contribute to explaining individual variability in later reading achievement.

## Discussion

The aim of this study was to explore the domain specificity of children’s individual differences in the size of several numerical effects (NDE, CE and SE) during intermediate grades of elementary school. We first examined the associations of the numerical effects with contemporaneous cognitive and linguistic variables, as well as with mathematics and reading skills, and then we tested, with hierarchical regression analyzes, whether numerical effects predict academic skills such as mathematics and reading one year later, after controlling for the effects of other predictor variables. The results showed that the size of SE in intermediate grades was a significant domain-specific predictor of mathematics fluency and also curricular mathematics achievement, but not reading skills, assessed at the end of elementary school. Furthermore, the size of CE also predicted fluency in calculation, although this association only approached significance. 

 This finding makes sense given that mathematics fluency and subitizing require quick recognition and combination of small magnitudes [19]. One possibility is that children having a strong foundation for subitizing and mapping the corresponding quantities are at an advantage in learning number facts. This explanation is in agreement with the point of view of Gallistel and Gelman [38] who consider that subitizing involves the use of a fast preverbal estimation of numerosities and the mapping from the resulting magnitudes to number words in order to rapidly generate the number words for small numerosities. On the other hand, the retrieval of the number facts is mediated via the inverse mappings from verbal and written numbers to preverbal magnitudes and the use of these magnitudes to find the appropriate cells in the tabular arrangements of the answers. This may explain the smaller subitizing range of children with mathematics disability found by Koontz and Berch [39]; it may also explain the common finding that children with mathematics disability have difficulties learning number facts [40]. It is noteworthy that the domain-specific relationship found for subitizing and fluency is generalized to other mathematical domains in which small numerosities are embedded. In line with this result, Fuchs and colleagues [19] found that precise representations of small quantities was uniquely predictive of mathematics fluency and word-problem tasks that change, combine, compare and equalize numerical relationships.

As highlighted in the Method section, one such common process used in both the enumeration task and the mathematics tasks, is the speed of processing. Thus, it could be argued that the predictive value of the SE in later mathematics skills is simply due to both kinds of tasks being speeded. However, by using each child’s RT to numerosity 1 as a baseline, we were able to calculate the SE as a ratio accounting for individual differences in RT. Moreover, our regression analysis demonstrated that the SE explained a significant amount of variance in mathematics outcomes after controlling individual differences in general processing speed.

Previous longitudinal studies have found that processing small numerosities is closely related to early mathematics learning [8,41,42], confirming that subitizing and counting constitute domain-specific foundational skills on which the formally acquired mathematics knowledge is built. As a novelty, the present research illustrates that mechanisms specialized in recognizing, representing and mentally manipulating small numerosities are not only “start –up” tools for the acquisition of mathematical competence during the first years of formal education, instead, they continue playing a domain-specific role on more sophisticated mathematics skills acquired in later grades, above and beyond phonological and lexical processing, nonverbal reasoning and general processing speed. 

This finding therefore encourages more empirical work to clarify the extent to which the Small Number System and mathematics knowledge are related [14,43]. Further research into this relationship that would examine a broader developmental age range in a longitudinal approach could reveal how subitizing modulates numerical symbolic acquisition from early to more advanced stages of mathematics knowledge above and beyond domain-general cognitive and linguistic mechanisms. On the other hand, it is known that the size of the subitizing effect decreases over developmental time [15]. However, it is unclear if this reduction simply reflects developmental changes in domain-general speed of processing and whether it is specific to numerical compared with nonnumerical stimuli. To examine these open questions, children at different ages and adults could be tested with numerical and nonnumerical subitizing tasks controlling for a measure of processing speed. 

Contrary to previous reports [21,44], we found that NDE did not account for a significant proportion of the variance for mathematics fluency and achievement in mathematics curriculum. Since these studies included children younger than those recruited for the present study, a possible explanation to this is that the predictive value of NDE decreases in the course of mathematics acquisition. Several authors [45,46] reported that the NDE decreases from kindergarten to fourth grade, with minor changes occurring thereafter. In fact, Holloway and Ansari [21] found a significant correlation between NDE and mathematical competence in 6 year-old children but this association was not significant in 8 year-olds. 

Additionally, in our study word and pseudoword decoding tests also failed to explain additional variance of mathematics fluency and other mathematics skills above and beyond general cognitive and numerical mechanisms. However, there is much support for the relationship between phonological awareness and mathematics achievement (see [47]). The triple-code hypothesis [48] states that language itself is needed to construct concepts of exact numbers greater than four, and several authors [40,49,50] argue that phonological awareness reflects the ability to differentiate between meaningful segments of language and to manipulate them, and this should consequently facilitate the differentiation and manipulation of single words in the number word sequence. However, some previous reports are in line with our finding; for instance, Durand and colleagues [51] found that phoneme deletion was a unique predictor of individual differences in reading but did not predict subsequent arithmetic skills. Moreover, Jordan and colleagues [12] reported that although basic reading proficiency was a strong predictor of mathematics achievement, it did not predict mathematics achievement above and beyond BNC. In another study, Fuchs and colleagues [17] reported that phonological processing (measured by rapid digit naming, first sound matching, and last sound matching) was a unique determinant of fact fluency, but did not predict other aspects of mathematics performance (e.g., story problems). In a subsequent study [52], the authors reported similar results when phonological processing was measured by phonological decoding of pseudowords. 

Just as interesting, besides the different contributions of SE and CE to each mathematics outcome after controlling for the effects of general cognitive skills, we found that the inclusion of domain-general predictors, in particular nonverbal reasoning, resulted in a 10% increase in R^2^ for predicting fluency in calculation, compared to a substantial increase (46%) in the accounted variance in mathematics tasks based on curriculum. These findings lend support to the notion that fluency in calculation and curricular achievement may represent distinct domains of mathematical cognition. In line with this assumption, Fuchs and colleagues [53] found that children situated at the lower-end of the performance range for calculation tasks exhibited distinctive cognitive profiles compared with children at the lower-end of the performance range for verbal problem-solving; whereas Hart and colleagues [54] demonstrated that mathematics problem solving has different genetic and environmental influences compared with fluency in calculation. 

In summary, in the present research, we find substantial evidence supporting that the domain-specific capacities, specifically subitizing and to a lower extent counting, are significantly related to more sophisticated mathematics skills acquired at the end of elementary school above and beyond domain-general abilities. This finding contrasts with proposals that the core numerical competencies measured by enumeration will bear little relationship to mathematics achievement. As practical implication, the present research supports the importance of training low-level numerical processing for enhancing mathematical competence in typically developed children even in later grades of elementary school.

Finally, certain considerations should be taken into account for interpreting the findings of the present study. Firstly, our sample size was modest. A small sample size makes it difficult to detect differences when following up significant multivariate interactions. However, power analyses were referred systematically in the Results Section to assist readers in evaluating whether power is a concern. This is particularly important as the conclusions are based partially on nonsignificant findings in certain relationships. Secondly, our measures of mathematics outcomes came from self-constructed tests without standardization. Although, this could be a partial limitation considering that the analysis was based on individual differences rather than on classifying children according to performance. 

## References

[1] Spelke ES, Kinzler KD (2007) Core knowledge. Dev Sci 10: 89-96. doi:10.1111/j.1467-7687.2007.00569.x. PubMed: 17181705. 17181705

[2] Carey S (2011) Précis of the origin of concepts. Behav Brain´s Sciences 34: 113-167. doi:10.1017/S0140525X10000919. PMC3489495 21676291

[3] Feigenson L, Dehaene S, Spelke E (2004) Core systems of number. Trends Cogn Sci 8: 307-314. doi:10.1016/j.tics.2004.05.002. PubMed: 15242690. 15242690

[4] Jordan NC, Glutting J, Ramineni C (2010) The importance of number sense to mathematics achievement in first and third grades. Learn Individ Differ 20: 82-88. doi:10.1016/j.lindif.2009.07.004. PubMed: 20401327. 20401327 PMC2855153

[5] Karmiloff-Smith A (1998) Development itself is the key to understanding developmental disorders. Trends Cogn Sci 2: 389-398. doi:10.1016/S1364-6613(98)01230-3. PubMed: 21227254. 21227254

[6] Jordan NC, Levine SC (2009) Socioeconomic variation, number competence, and mathematics learning difficulties in young children. Dev Disabilities Res Rev 15: 60-68. doi:10.1002/ddrr.46. PubMed: 19213011. 19213011

[7] Geary DC (2011) Consequences, characteristics, and causes of mathematical learning disabilities and persistent low achievement in mathematics. J Dev Behav Pediatr 32: 250-263. doi:10.1097/DBP.0b013e318209edef. PubMed: 21285895. 21285895 PMC3131082

[8] LeFevre JA, Skwarchuk SL, Smith-Chant BL, Bisanz J, Kamawar D et al. (2010) Pathways to mathematics: Longitudinal predictors of Performance. Child Dev 81: 1753-1767. doi:10.1111/j.1467-8624.2010.01508.x. PubMed: 21077862. 21077862

[9] Methe S, Hintze J, Floyd R (2008) Validation and decision accuracy of early numeracy skill indicators. Sch Psychol Rev 37: 359-373.

[10] Aunola K, Leskinen E, Lerkkanen MK, Nurmi JE (2004) Developmental dynamics of math performance from preschool to grade 2. J Educ Psychol 96: 699-713. doi:10.1037/0022-0663.96.4.699.

[11] Passolunghi MC, Vercelloni B, Schadee H (2007) The precursors of mathematics learning: Working memory, phonological ability and numerical competence. Cogn Dev 22: 165-184. doi:10.1016/j.cogdev.2006.09.001.

[12] Jordan NC, Kaplan D, Nabors OL, Locuniak MN (2006) Number sense growth in kindergarten: A longitudinal investigation of children at risk for mathematics difficulties. Child Dev 77: 153-175. doi:10.1111/j.1467-8624.2006.00862.x. PubMed: 16460531. 16460531

[13] Halberda J, Mazzocco MMM, Feigenson L (2008) Individual differences in nonverbal number acuity predict maths achievement. Nature 455: 665-668. doi:10.1038/nature07246. PubMed: 18776888. 18776888

[14] Piazza M (2010) Neurocognitive start-up tools for symbolic number representations. Trends Cogn Sci 14: 542-551. doi:10.1016/j.tics.2010.09.008. PubMed: 21055996. 21055996

[15] Reeve R, Reynolds F, Humberstone J, Butterworth B (2012) Stability and change in markers of core numerical competencies. J Exp Psychol Gen 141: 649-666. doi:10.1037/a0027520. PubMed: 22409662. 22409662

[16] Bull R, Johnston RS (1997) Numerical magnitude representations influence arithmetic learning. Child Dev 65: 1-24. 10.1111/j.1467-8624.2008.01173.x 18717904

[17] Fuchs LS, Compton DL, Fuchs D, Paulsen K, Bryant JD et al. (2005) The prevention, identification, and cognitive determinant of math difficulty. J Educ Psychol 97: 493-513. doi:10.1037/0022-0663.97.3.493.

[18] Hitch GJ, McAuley E (1991) Working memory in children with specific arithmetical learning disabilities. Br J Psychol 82: 375-386. doi:10.1111/j.2044-8295.1991.tb02406.x. PubMed: 1954527. 1954527

[19] Fuchs LS, Geary DC, Compton DL, Fuchs D, Hamlett CL et al. (2010) The contributions of numerosity and domain-general abilities to school readiness. Child Dev 81: 1520-1533. doi:10.1111/j.1467-8624.2010.01489.x. PubMed: 20840238. 20840238 PMC2941220

[20] Jordan NC, Montani TO (1997) Cognitive arithmetic and problem solving: A comparison of children with specific and general mathematics difficulties. J Learn Disabilities 30: 624-634. doi:10.1177/002221949703000606. PubMed: 9364900. 9364900

[21] Holloway ID, Ansari D (2009) Mapping numerical magnitudes onto symbols: The numerical distance effect and individual differences in children's mathematics achievement. J Exp Child Psychol 103: 17-29. doi:10.1016/j.jecp.2008.04.001. PubMed: 18513738. 18513738

[22] Geary DC, Hoard MK, Hamson CO (1999) Numerical and arithmetical cognition: patterns of functions and deficits in children at risk for mathematical disability. J Exp Child Psychol 74: 213-239. doi:10.1006/jecp.1999.2515. PubMed: 10527555. 10527555

[23] Reigosa-Crespo V, Valdés-Sosa M, Butterworth B, Estévez N, Rodríguez M et al. (2012) Basic numerical capacities and prevalence of developmental dyscalculia: The Havana survey. Dev Psychol 48: 123-135. doi:10.1037/a0025356. PubMed: 21910533. 21910533

[24] Desoete A, Ceulemans A, Roeyers H, Huylebroeck A (2009) Subitizing or counting as possible screening variables for learning disabilities in mathematics education or learning? Educ Res 4: 55-66.

[25] Landerl K, Kölle C (2009) Typical and atypical development of basic numerical skills in elementary school. J Exp Child Psychol 103: 546-565. doi:10.1016/j.jecp.2008.12.006. PubMed: 19254797. 19254797

[26] Schleifer P, Landerl K (2010) Subitizing and counting in typical and atypical development. Dev Sci, 13: 1-12. PubMed: 20121858. 22213901 10.1111/j.1467-7687.2010.00976.x

[27] National Mathematics Advisory Panel (2008) Foundations for success: Final report of the National Mathematics Advisory Panel. Washington, DC: United States Department of Education.

[28] Goldman SR, Pellegrino JW, Mertz DL (1998) Extended practice of addition facts: Strategy changes in learning-disabled students. Cogn Instruction 5: 223-265.

[29] Raven JC, Court JH, Raven J (1986) Manual for Raven progressive matrices and vocabulary scales. London: Lewis Publishing House.

[30] Butterworth B (2003) Dyscalculia screener. London, England: NFERNelson.

[31] Reigosa-Crespo V, Pérez-Abalo MC, Manzano M, Antelo JM (1994) SAL: Sistema automatizado para explorar la lectura en escolares de habla hispana. Rev Lat Pensa Lenguaje 2: 134 - 141.

[32] Girelli L, Lucangeli D, Butterworth B (2000) The development of automaticity in accessing number magnitude. J Exp Child Psychol 122: 104 -122. PubMed: 10788305. 10.1006/jecp.2000.2564 10788305

[33] Landerl K, Bevan A, Butterworth B (2004) Developmental dyscalculia and basic numerical capacities: A study of 8-9-year-old students. Cognition 93: 99-125. doi:10.1016/j.cognition.2003.11.004. PubMed: 15147931. 15147931

[34] Hammill DD, Wiederholt JL, Allen EA (2006) Test of silent contextual reading fluency. Austin, TX: PRO-ED.

[35] Dehaene S, Cohen L (1994) Dissociable mechanisms of subitizing and counting: Neuropsychological evidence from simultanagnosis patients. J Exp Psychol Hum Percept Perform 20: 958-975. doi:10.1037/0096-1523.20.5.958. PubMed: 7964531. 7964531

[36] Mandler G, Shebo BJ (1982) Subitizing: An analysis of its component processes. J Exp Psychol Gen 111: 1-21. doi:10.1037/0096-3445.111.1.1. PubMed: 6460833. 6460833

[37] Trick LM, Pylyshyn ZW (1993) What enumeration studies can show us about spatial attention: evidence for limited capacity preattentive processing. J Exp Psychol Hum Percept Perform 19: 331-351. doi:10.1037/0096-1523.19.2.331. PubMed: 8473843. 8473843

[38] Gallistel CR, Gelman R (1992) Preverbal and verbal counting and computation. Cognition 44: 43-74. doi:10.1016/0010-0277(92)90050-R. PubMed: 1511586. 1511586

[39] Koontz KL, Berch DB (1996) Identifying simple numerical stimuli: Processing inefficiencies exhibited by arithmetic learning disabled children. Math Cogn 2: 1-24. doi:10.1080/135467996387525.

[40] Geary DC (1993) Mathematical disabilities: Cognitive, neuropsychological, and genetic components. Psychol Bull 114: 345-362. doi:10.1037/0033-2909.114.2.345. PubMed: 8416036. 8416036

[41] Kroesbergen EH, Van Luit JEH, Van Lieshout EC, Loosbroek EV, Van de Rijt BA (2009) Individual differences in early numeracy. The role of executive functions and subitizing. J Psychoeducational Assess 27: 226 -236. doi:10.1177/0734282908330586.

[42] Krajewski K, Schneider W (2009) Early development of quantity to number-word linkage as a precursor of mathematical school achievement and mathematical difficulties: Findings from a four-year longitudinal study. Learn Instruction 19: 513-526. doi:10.1016/j.learninstruc.2008.10.002.

[43] Butterworth B (2010) Foundational numerical capacities and the origins of dyscalculia. Trends Cogn Sci 14: 534-541. doi:10.1016/j.tics.2010.09.007. PubMed: 20971676. 20971676

[44] Smedt BD, Verschaffel L, Ghesquière P (2009) The predictive value of numerical magnitude comparison for individual differences in mathematics achievement. J Exp Child Psychol 103: 469-479. doi:10.1016/j.jecp.2009.01.010. PubMed: 19285682. 19285682

[45] Duncan EM, McFarland CE (1980) Isolating the effects of symbolic distance and semantic congruity in comparative judgments: An additive factors analysis. Mem Cogn 8: 612-622. doi:10.3758/BF03213781. 6163942

[46] Sekuler R, Mierkiewicz D (1977) Children's judgements of numerical inequality. Child Dev 48: 630-633. doi:10.2307/1128664.

[47] Simmons FR, Singleton C (2008) Do weak phonological representations impact on arithmetic development? A review of research into arithmetic and dyslexia. John Wiley & Sons, Ltd. pp. 77-94. 10.1002/dys.341 17659647

[48] Dehaene S, Piazza M, Pinel P, Cohen L (2003) Three parietal circuits for number processing. Cogn Neuropsychol 3: 487-506. PubMed: 20957581. 10.1080/02643290244000239 20957581

[49] Kulak AG (1993) Parallels between math and reading disability: Common issues and approaches. J Learn Disabilities 26: 666-673. doi:10.1177/002221949302601004. PubMed: 8151206. 8151206

[50] Fuson CK (1988) Children’s counting and concepts of number. New York: Springer-Verlag.

[51] Durand M, Hulme C, Larkin R, Snowling M (2005) The cognitive foundations of reading and arithmetic skills in 7- to 10-year-olds. J Exp Child Psychol 91: 113-136. doi:10.1016/j.jecp.2005.01.003. PubMed: 15890173. 15890173

[52] Fuchs LS, Fuchs D, Compton DL, Powell SR, Seethaler PM et al. (2006) The cognitive correlates of third-grade skill in arithmetic, algorithmic computation, and arithmetic word problems. J Educ Psychol 98: 29-43. doi:10.1037/0022-0663.98.1.29.

[53] Fuchs LS, Fuchs D, Craddock C, Hollenbeck KN, Hamlett CL et al. (2008) Effects of small group tutoring with and without validated classroom instruction on at-risk students´ math problem solving. J Educ Psychol 100: 491-509. doi:10.1037/0022-0663.100.3.491. PubMed: 19122881. 19122881 PMC2536765

[54] Hart SA, Petrill SA, Thompson LA (2010) A factorial analysis of timed and untimed measures of mathematics and reading abilities in school aged twins. Learn Individ Differ 20: 63-69. doi:10.1016/j.lindif.2009.10.004. PubMed: 20161680. 20161680 PMC2821061

